# Support for calcium channel gene defects in autism spectrum disorders

**DOI:** 10.1186/2040-2392-3-18

**Published:** 2012-12-15

**Authors:** Ake Tzu-Hui Lu, Xiaoxian Dai, Julian A Martinez-Agosto, Rita M Cantor

**Affiliations:** 1 Department of Human Genetics, David Geffen School of Medicine, University of California at Los Angeles, Los Angeles, CA 90024-7088, USA; 2 Center for Neurobehavioral Genetics, David Geffen School of Medicine, University of California at Los Angeles, Los Angeles, USA

**Keywords:** Autism spectrum disorders, Calcium channel genes, Common variants, Imputed SNPs, Association studies

## Abstract

**Background:**

Alternation of synaptic homeostasis is a biological process whose disruption might predispose children to autism spectrum disorders (ASD). Calcium channel genes (CCG) contribute to modulating neuronal function and evidence implicating CCG in ASD has been accumulating. We conducted a targeted association analysis of CCG using existing genome-wide association study (GWAS) data and imputation methods in a combined sample of parent/affected child trios from two ASD family collections to explore this hypothesis.

**Methods:**

A total of 2,176 single-nucleotide polymorphisms (SNP) (703 genotyped and 1,473 imputed) covering the genes that encode the α_1_ subunit proteins of 10 calcium channels were tested for association with ASD in a combined sample of 2,781 parent/affected child trios from 543 multiplex Caucasian ASD families from the Autism Genetics Resource Exchange (AGRE) and 1,651 multiplex and simplex Caucasian ASD families from the Autism Genome Project (AGP). SNP imputation using IMPUTE2 and a combined reference panel from the HapMap3 and the 1,000 Genomes Project increased coverage density of the CCG. Family-based association was tested using the FBAT software which controls for population stratification and accounts for the non-independence of siblings within multiplex families. The level of significance for association was set at 2.3E-05, providing a Bonferroni correction for this targeted 10-gene panel.

**Results:**

Four SNPs in three CCGs were associated with ASD. One, *rs10848653*, is located in *CACNA1C*, a gene in which rare *de novo* mutations are responsible for Timothy syndrome, a Mendelian disorder that features ASD. Two others, *rs198538* and *rs198545*, located in *CACN1G*, and a fourth, *rs5750860*, located in *CACNA1I*, are in CCGs that encode T-type calcium channels, genes with previous ASD associations.

**Conclusions:**

These associations support a role for common CCG SNPs in ASD.

## Background

Autism spectrum disorders (ASD) are a group of neurodevelopmental traits characterized by behavioral symptoms in three domains: deficits in communication skills, deficits in social skills, and the presence of restricted repetitive behaviors [1]. ASD prevalence is currently estimated at 1/88 children (http://www.cdc.gov/media/releases/2012/p0329_autism_disorder.html), with a 4:1 ratio of boys to girls [2]. A recent twin study indicates that ASD is heritable, but its etiology is likely to include both genetic and environmental factors and their interactions [3]. All studies indicate that the etiology of ASD is likely to be very heterogeneous, and most predisposing genetic and environmental risk factors are currently unidentified. Recent whole-genome exon sequencing studies of ASD samples estimate that as many as 100 to 1,000 genes may be involved [4].

The current hunt for ASD genes is focused on whole exome sequencing to identify rare *de novo* mutations in simplex families [5,6]. In contrast, we hypothesize that targeted association analyses of common variants in ASD candidate genes can provide complementary information that is valuable. We report herein an association study that examines the family of calcium channel genes (CCG) that is supported by: (1) biologic insights into the roles of these genes in the brain [7]; (2) evidence derived from a Mendelian disorder that features ASD [8]; and (3) several previous more limited linkage and association studies of ASD.

Biological support for a role of CCG in autism derives from their role in the brain [7]. During depolarization, voltage-gated Ca^2+^ channels mediate influx of calcium into neurons, eliciting a number of calcium-modulated functions including neurotransmitter release, intracellular signaling, and gene transcription. The channels are composed of a central pore-forming α_1_ subunit that interacts with other auxiliary and regulatory subunits: α_2_δ_,_ β, and γ. The α_1_ subunit, which is the largest, forms the Ca^2+^ channel selective ‘pore’ that determines calcium selectivity. Identified by their calcium current types, the 10 α_1_ subunits are clustered into three subfamilies denoted by Ca_v_1, Ca_v_2, and Ca_v_3, respectively. Table 1 lists the gene names, their channel names, the types of calcium currents, and their gene expression patterns [9]. We reasoned that given the central role for α_1_ subunits in forming the pore essential for calcium channel function, variants in this group of CCG might affect neuronal calcium entry and contribute to ASD susceptibility. A survey of the expression pattern for each of the subunits in human brain demonstrates that each is present in neurons of the cerebral cortex, supporting their relevance as candidates for analysis in ASD [10].

**Table 1:** **10 α**_**1 **_**subunit calcium channel genes tested for association with ASD**

**Gene symbol (Chrom band)**	**Channel**	**Current type**^**a**^	**Most dominant brain expressed region**^**a**^	**Cells and tissues with gene expression in addition to brain**
**Ca**_**v **_**1 subfamily**				
CACNA1S (1q32)	Ca_v_1.1	L	MD	Skeletal muscle; transverse tubules
CACNA1C (12p13.3)	Ca_v_1.2	L	MD	Cardiac myocytes; smooth muscle myocytes; endocrine cells; neurons
CACNA1D (3p14.3)	Ca_v_1.3	L	NCX	Endocrine cells; neurons; cardiac cells and pacemaker cells; cochlear hair cells
CACNA1F (Xp11.23)	Ca_V_1.4	L	Amygdala	Retina; spinal cord; adrenal gland; mast cells
**Ca**_**v **_**2 subfamily**				
CACNA1A (19p13)	Ca_v_2.1	P/Q	CBC	Neurons
CACNA1B (9q34)	Ca_v_2.2	N	MD	Neurons
CACNA1E (1q25-q31)	Ca_v_2.3	R	Striatum	Neurons
**Ca**_**v **_**3 subfamily**				
CACNA1G (17q21)	Ca_v_3.1	T	MD	Neurons; smooth muscle myocytes
CACNA1H (16p13.3)	Ca_v_3.2	T	Striatum	Neurons; cardiac and smooth muscle myocytes
CACNA1I (22q13.1)	Ca_v_3.3	T	NCX	Neurons

^a^Current types were defined based on different properties in biophysical and pharmacological analysis.

*CBC *Cerebellar cortex; *L *Long lasting; *MD *Mediodorsal nucleus of the thalamus; *N *Neither long nor transient lasting; *NCX*, Areas of neocortex; *P*, Cerebellar Purkinje cell; *Q *Indicating different toxic sensitivity and inactivation rate from the P-type in α_1A _subunit [11]; *R* Resistent; *T* Transient lasting.

The most salient prior genetic evidence implicating CCG in ASD comes from a *CACNA1C* gene mutation that results in Timothy syndrome (TS), a Mendelian disorder with delayed repolarization of the heart following a heartbeat [12]. TS features ASD along with deficits in language and social development [13]. It is caused by a *de novo* missense mutation in the eighth exon of *CACNA1C* that encodes the alpha 1C subunit proteins of an L-type voltage-gated calcium (Ca^2+^) channel (high voltage activation and slow voltage-dependent inactivation with long-lasting currents). Additional genetic support of a role for CCG in ASD comes from the association with ASD of a single-nucleotide polymorphism (SNP) in the *CACNA1G* gene encoding a T-type Ca^2+^ channel subunit [14] (transient duration of opening) detected in an analysis of parent/child ASD affected trios from 284 nuclear multiplex families with only affected boys from the Autism Genetics Research Exchange (AGRE) collection. *CACNA1G* is located within a chromosome 17-linked region (17q11-21) that has been identified and formally replicated in families with only affected boys [15,16]. Activities of T-type Ca^2+^ channels are associated with neuronal firing in the brain [17]. An additional T-type CCG, *CACNA1H*, has been implicated in ASD through previous gene sequencing studies. Heterozygous missense mutations were identified in six out of 461 individuals with ASD from the AGRE panel [18]. This gene is expressed in many regions of the brain that exhibit abnormal sizes in individuals with ASD [19]. The mutations identified alter Ca_v_3.2 channel function by decreasing voltage sensitivity, slowing channel activation, and disrupting channel inactivation, causing sustained large calcium currents [12].

Recently, exon sequencing has identified *de novo* mutations in two other CCGs, *CACNA1D* and *CACNA1E*, in a sample of 209 sporadic ASD families that have no previous history of ASD or its related phenotypes [20]. *CACNA1I* was previously implicated in ASD by a GWAS analysis that applied a noise reduction approach to boost statistical power (GWAS-NR) in a combined sample of 597 Caucasian ASD families collected by the Hussman Institute for Human Genomics (HIHG) and 696 AGRE multiplex families [21]. A haplotype block in *CACNA1I* was associated with a *P* value of 1.8E-05.

In the current study we assess the potential role of CCG in ASD by focusing on 10 genes that encode α_1_ subunits. A dense panel of SNPs is tested for association with ASD in the combination of two study samples ascertained for families with ASD with genotypes available through ongoing studies of accumulated and public GWAS data: 543 multiplex ASD families from the AGRE repository [22] and 1,651 families from the Autism Genome Project (AGP) [23]. SNP coverage of the 10 CCGs has been extended by SNP imputation, and association has been tested using the Family Based Association Test (FBAT) software. Using this approach, the association analysis is not vulnerable to the effects of population stratification and is corrected for the non-independence of sibling pairs. Four SNPs in *CACN1C*, *CACNA1I*, and *CACN1G* meeting the Bonferroni corrected level of significance were considered to be associated with ASD, further supporting the relevance of CCG to ASD.

## Methods

### Overall study design

Using GWAS data available to interested researchers and methods of imputation, a dense panel of 2,176 pruned common SNPs (703 called and 1,473 imputed) in 10 CCGs was tested for association with ASD using the FBAT software that corrects for population stratification and non-independence of parent/child trios within nuclear families. With this approach, each SNP is tested for a transmission ratio that differs significantly from its expected 50%. The study sample consisted of 2,781 Caucasian parent/child trios, where 1,103 are from 543 AGRE families and 1,678 are from 1,651 AGP families. Prior to analysis, 10% to 15% of the families where one or both parents were not Caucasian were identified using multidimensional scaling analysis and removed to reduce genetic heterogeneity that could be attributable to race. SNP imputation was conducted separately in the AGRE and AGP panels using the IMPUTE2 software and a combined reference panel from the HapMap3 and the 1,000 Genomes Project. Allele frequencies and patterns of linkage disequilibrium were estimated within each sample for the imputed SNPs. These were compared for consistency. A Bonferroni correction for the 2,176 SNPs tested was used to define a level of significance of *P* <2.3E-05 that was used for association, as we had not examined the association of any of the genotyped SNPs in either sample prior to the analyses reported herein.

### Study sample and genotyping: AGRE

The AGRE repository is described fully in [22]. It is composed of nuclear families ascertained for two or more children with ASD identified by the Autism Diagnostic Instrument Revised (ADIR) [24]. Monozygotic twins and children with non-idiopathic ASD such as fragile X, abnormal brain imaging, abnormal karyotype, neurogenetic disorder, and perinatal insults were excluded from the panel for the current analyses. Both parents and the children of 731 families had GWAS genotyping performed on two platforms: 86% on the Illumina HumanHap 550 BeadChip platform and 14% on the HumanOmni1-Quad BeadChip platform. Quality control (QC) of SNPs included removing those with Hardy-Weinberg Equilibrium *P* <0.0001, a Mendelian error rate >0.1, or a minor allele frequency (MAF) <0.01. Multidimensional scaling analysis [25] was performed to identify the founder ancestry of the parents, resulting in 543 multiplex Caucasian families (1,103 trios) for association analysis. Regulatory review, approval, and oversight of AGRE's human subject research is provided by Western IRB, an AAHRPP-accredited Independent Review Board located in Olympia, WA (AGRE website).

### Study sample: AGP

AGP families were ascertained for children with ASD and collected from more than 50 centers in North America and Europe and combined into a single panel for genotyping and GWAS analysis. The individual study samples that comprise the AGP each comply with the current laws of the country in which the samples were collected. The AGRE families that are part of AGP panel have been excluded from the AGP for the work reported here. Children with ASD are identified by positive results on ADI-R [24] and the Autism Diagnostic Observation Schedule (ADOS) [26]. A total of 1,884 AGP trios and nuclear families recruited from Phase 2-Stage1 were genotyped using the Illumina Human 1M-single Infinium BeadChip platform. Similar to the AGRE panel, non-idiopathic ASD children have been excluded from analysis. SNPs were filtered using the same QC criteria. Multidimensional scaling analysis (MDS), equivalent to that performed for the AGRE families, identified 1,651 Caucasian families (1,678 trios) to be combined with the 543 AGRE families for association analysis.

### Multidimensional scaling: identifying Caucasian families within AGRE and the AGP

MDS was conducted using the PLINK software to identify the Caucasian founders separately in the AGRE and AGP samples. The genetic similarity between N founders was estimated based on identity by state (IBS) marker concordance at every fifth marker on the autosomal chromosomes. This yielded an N by N similarity matrix. Principal components analysis was performed to transform the estimated matrix in order to project the greatest amount of IBS information in a two dimensional plot, geometrically clustering the founders with the same genetic ancestry. Eighty percent of the AGRE founders were identified as Caucasians and 93% of the AGP founders were categorized into the AGRE cluster classified as Caucasian. Both parents had to be classified into this cluster for the family to be retained in the analysis. A total of 543 AGRE (1,103 trios) families and 1,651 AGP (1,678 trios) were classified as Caucasian.

### SNP imputation in the 10 CCG: methods and quality control

Imputation of SNP genotypes is based on genotyped SNPs that are IBS where two unrelated individuals share short stretches of their haplotypes from their common ancestors. Current algorithms for genotype imputation are based on hidden Markov models (HMM) and include Beagle [27], MaCH [28], IMPUTE, and IMPUTE2 [29-31]. For the work reported here, imputation was conducted using IMPUTE2 [30,31] with the CEU (Utah residents with northern and western European ancestry) reference panel. For the reference data, a combined panel from the HapMap 3 and 1,000 Genomes projects were used [32,33]. All have very high accuracy as indicated by concordance rates of between 94% and 97% for called and imputed genotypes when masking those that were called [31]. For haplotype reconstruction, HMM are used to infer the haplotype phase and impute the missing genotypes. The models include a matrix of transition probabilities to allow for the occurrence of recombination between adjacent markers and a matrix of emission probabilities to mimic the effect of mutation.

To insure the availability of only high quality imputed genotypes for analyses with IMPUTE2, the developers of this program filtered the reference Hapmap3 and 1,000 Genomes SNP data with quality control measures and removed those SNPs with problems as described on their website (http://mathgen.stats.ox.ac.uk/impute/data_download_1000G_pilot_plus_hapmap3.html). The effect size was set at 2,000 (−*Ne* option) to scale the recombination rates in the HMM. Each imputed marker was assessed by an information measure that ranged from 0 to 1. The value of one is achieved when the information from imputed genotypes equals the information one would expect if the alleles are genotyped and sampled from the population. To assign the imputed genotypes for each individual, IMPUTE2 outputs three posterior probability scores corresponding to three possible genotypes and assigns the one with the highest score. Imputed SNPs and their genotypes were accepted at a measure >0.4 and a probability score >0.9. SNP pruning was implemented under PLINK to decrease the redundancy by using the linkage disequilibrium threshold of R^2^ ≥0.99. MaCH was used to validate the imputed SNPs that were found to be associated with ASD. Imputation of genotypes performed well in both the AGRE and AGP samples with an overall 95% concordance rate. After SNP pruning of 3,675 markers, 2,176 SNPs (703 called, 1,473 imputed) remained for association analysis.

For the associated SNPs that were imputed, an additional quality check was used. The SNP allele frequencies were estimated separately in the AGP and AGRE samples. The frequency estimates were the same for each SNP indicating that if there were an error in imputation it was the same in both samples. Then linkage disequilibrium was estimate for adjacent genotyped SNPs for the two imputed associated SNPs in AGP and AGRE separately. These estimates are each very close in both samples.

### Association analysis: family based association test (FBAT) in the combined AGRE and AGP sample

The FBAT software was used to test for association of ASD and the panel of 2,176 SNPs. Genetic effects were assumed to be additive, and the -e option was used to adjust for the correlation between sibling marker genotypes in multiplex families [34]. Markers on X-chromosome for the *CACNA1F* gene were also tested using FBAT [35]. The level of significance, *P* <2.3E-05, was based on a Bonferroni correction for testing 2,176 SNPs.

### Copy number variation, transcription factor binding site, and brain expression pattern analysis

The University of California Santa Cruz Genome browser (hg19) was used to identify chromatin immunoprecipitation sites and copy number variations and their associated phenotypic findings reported to the DECIPHER and ISCA consortia including the loci identified in this study. Transcription factor binding site consensus sequences were determined using Tfsitescan (http://www.ifti.org/cgi-bin/ifti/Tfsitescan.pl). Brain expression patterns were assessed for each gene using the Human Brain Transcriptome Atlas (HBT; http://hbatlas.org/pages/hbtd) and GENSAT databases (http://www.gensat.org/search.jsp). All of the 10 CCG were expressed in brain regions based on the spatiotemporal gene expression data provided by human brain transcriptome (HBT, http://hbatlas.org/). Their most dominant expression regions in brain (Table 1) were based on the data at the age 3 time-point. Eight of them, except *CACNA1S* and *CACNA1F*, were determined as highly expressed in specific brain regions by considering a log_2_ of signal intensity >6 [10]. The regions of enriched gene expression were confirmed with the mouse *in situ* expression in GENSAT.

## Results

Table 2 reports the three CCGs containing four SNPs that pass the criterion for association. Most encouraging is the association of *rs108486653*, an imputed SNP within the *CACN1C* gene which encodes Ca_v_1.2, a subunit of a calcium channel that is expressed predominantly in neurons and cardiac pacemaker cells. A genetic overlap between ASD, bipolar disorder, and schizophrenia has been hypothesized [36], and *CACNA1C* has previously been associated with bipolar disorder in two GWAS [37,38]. Most salient, however, is that *de novo* mutations in this gene cause Timothy syndrome that has ASD as a prominent feature. While that very rare causal variant may not be present in these samples, this association indicates that a more common allele with a frequency of 0.32 in this sample may tag a variant that contributes to the risk for ASD.

**Table 2:** Calcium channel genes associated with ASD

**CCG**	**Associated SNP**	**Chromosome/Basepair**^**b**^	**Minor/Major allele**	**MAF**	**T:U**^**c**^	***P *value**
**Ca**_**v**_**1.2 CACNA1C**	rs10848653^a^	12/2,358,200	G/**A**	0.32	825:643	1.3E-06
**Ca**_**v**_**3.1 CACNA1G**	rs198538	17/45,997,692	T/**C**	0.20	980:779	1.5E-06
	rs198545	17/46,000,610	T/**G**	0.06	359:247	1.8E-05
**Ca**_**v**_**3.3 CACNA1I**	rs5750860^a^	22/38,355,980	T/**C**	0.18	703:535	7.4E-06

^a^Imputed SNP.

^b^Human genome assembly HG18.

^c^T:U, major allele #Transmitted: # Untransmitted.

*MAF *Minor allele frequency.

The ratio of transmissions to non-transmissions of the major allele in this sample of more than 2,700 parent/child trios, with 5,400 potentially heterozygous and informative parents is 825:643. This SNP has been imputed, and since its information measure of nearly 0.9 indicates there is little uncertainty in the imputed genotypes, we are convinced that the imputed genotypes and the resulting association are quite accurate. Supporting this is a detectable Mendelian error rate of <2% in the AGRE sample and 0 in the AGP sample. The minor allele frequency and the pattern of linkage disequilibrium with its neighboring SNPs are consistent across the AGRE and AGP study samples where it was imputed separately, further supporting the accuracy of its imputed genotypes. This SNP is not within the eighth exon of *CACN1C*, where the Timothy syndrome mutation resides, but is within an intron of the gene.

**Figure 1:**
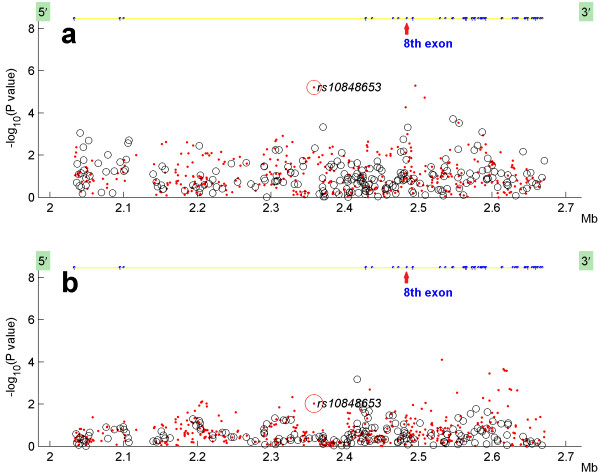
**Association analysis of genotyped and imputed SNPs in CACNA1C gene in (a) AGRE and (b) AGP samples. ***CACNA1C *gene SNP associations showing the relative locations of the Timothy Syndrome mutation and the SNP with the strongest association in the AGRE sample (upper panel) and the AGP sample (lower panel).

Figure 1 illustrates the association results of the imputed and genotyped SNPs within the *CACN1C* gene. Consistent with the view that simplex and multiplex families exhibit different genetic architecture, the results are stronger in the multiplex AGRE sample, which is more likely to have common associated SNPs than the mixed simplex and multiplex AGP families that are thought to have more rare variants with greater penetrance. While the associated SNP is not within exon 8, this exon shows more SNPs with what might be considered marginally significant results in both samples.

The next two associated SNPs reported in Table 2 are in *CACNA1G*. They have been genotyped in this study sample, and the genotypes have passed stringent quality control criteria and the allele frequencies and patterns of linkage disequilibrium with neighboring SNPs are consistent across the AGRE and AGP samples, providing us with confidence in this association. Although their allele frequencies differ, 0.20 and 0.06, they exhibit some degree of linkage disequilibrium, with each other (D’ = 0.83). Neither was associated in the previous study that implicated this gene, and the SNP implicated in that study is not associated here [14], However, this is a much larger study having a different design and method of analysis. *CACNA1G* encodes the Ca_v_3.1 Ca^2+^ channel subunit in the third CCG subfamily, a T-type Ca^2+^ channel, listed in Table 1. Interestingly, the *rs198538* SNP is located within a well-conserved portion of the genome that can be pulled down by Egr-1 chromatin immunoprecipitation [39,40].

The fourth associated SNP in Table 2 is *rs5750860* in *CACNA1I*, which is also a T-Type calcium channel gene with properties similar to those of *CACNA1G*. This SNP has been imputed, and again confidence in the genotypes arises from strong information measure, low rates of Mendelian errors of 2.3% in the AGRE sample and 0% in the AGP sample, and consistent allele frequencies and patterns of linkage disequilibrium with neighboring SNPs in these samples. Prior to this study, all three T-type CCG were implicated in ASD [14,18,21], and we thus provide additional supportive evidence. The unique features of this channel subtype may eventually help provide insight into the means by which the T-Type genes could predispose to ASD.

## Discussion

Prior to the availability and widespread adoption of GWAS technology, heritability estimates for complex traits and disorders were derived from phenotypic correlations in relative pairs (primarily twins) [41]. Heritability estimates have not been explained by the additive combinations of SNPs associated at the genome-wide significance level of 5.0E-8 for most traits [42]. In response, there has been a growing interest in incorporating GWAS SNPs that show some, but not a significant, association, into estimates of the genetic contributions to complex traits and disorders [21,43]. That is, investigators are hypothesizing that the ‘missing heritabilities’ for complex disorders might be found in the polygenic contributions of SNPs with positive associations that do not achieve a genome-wide level of significance because their effects are too small to provide adequate statistical power in the available study samples for their detection [44]. Studies applying the polygenic model now report evidence of missing heritability among the ‘non-significant associations’ [45]. Here we consider the same possibility of smaller undetected effects among the non-significant GWAS SNPs, but take a different approach to their detection and application to ASD. We focus on a specific set of genes that exhibit biological plausibility and support along with prior evidence of association with ASD. Ten CCG are tested using the best study samples and analytic approaches available for these endeavors. Two existing GWAS are combined into one sample, providing more than 2,700 parent/affected child trios for analysis, and SNP imputation is conducted to insure adequate coverage of the genes. Four associated SNPs in three CCG exceeded the level of significance.

Prior evidence for our focus on CCG derived from the ASD component of TS, which results from a *de novo* mutation in the eighth exon of a CCG, *CACNA1C*. We hypothesized that this genetic cause of a Mendelian form of ASD may be part of a family of genes that could predispose to the genetically complex idiopathic forms of ASD. To pursue this we tested the 10 CCGs that encode the largest subunits, α_1_, of 10 distinct Ca^2+^ channels. Biologically, these channels couple depolarization to a vast number of intracellular neuronal functions modulated by calcium, including signaling, gene transcription, and neurotransmitter release [46]. The α_1_ subunit contains both the voltage-sensing mechanism and forms the calcium-selective pores, mediating the calcium current [12,47]. Mutations in *CACNA1C* cause a significant increase in the sustained intracellular calcium rise, leading to changes in gene expression and altered neuronal differentiation, partly through changes in early growth response protein (Egr1) transcription factor levels [48]. The associated SNP we detected in *CACNA1C* may be tagging such a mutation or a variant with a lesser effect.

The *rs198538* SNP in the *CACNA1G* gene identified in this study is located within a portion of the genome that can be pulled down by Egr1 chromatin immunoprecipitation [39]. Its association with autism comes from its fellow family member, Egr2/krox20. Egr2 was identified as the most downregulated gene in a study that analyzed lymphoblastoid cell line gene expression among monozygotic autistic twin sets in the AGRE cohort [49]. Most interestingly, the *rs198538* SNP forms part of a consensus binding site for the transcription factor aryl hydrocarbon nuclear receptor translocator (Arnt), CA**C**GCW (Tfsitescan). Within this binding site, CA**C**GCACTG, the second C (underlined) is conserved across evolution and is polymorphic in the population, corresponding to the *rs198538* SNP. A genetic association has been found between the *ARNT2* gene and both autism and Asperger syndrome [50]. In addition, rare variants in the *ARNT2* gene have been identified in patients with ASD [6,51]. A member of the basic-helix-loop-helix (bHLH-PAS) superfamily of transcription factors, ARNT2 is highly expressed in brain [52] and forms complexes with hypoxia inducible factor (HIF1alpha) and the arylhydrocarbon receptor (AHR) to mediate neuronal responses to oxygen and xenobiotics [53]. While the ability of *ARNT2* to participate in AHR-mediated responses to xenobiotics is still under debate, this SNP variant and those in *ARNT2* may contribute to the environmental influences observed in ASD [54]. Furthermore, as in Timothy syndrome, excessive calcium-mediated signaling may underlie the brain pathology in carriers of common SNP or rare single nucleotide variants in calcium channels. The recent findings of a *de novo* synonymous variant in *CACNA1G* in an ASD proband from the Simons Simplex Collection [4] and missense mutations in *CACNA1H* in six of 461 individuals with ASD [18] further supports the potential for the genes identified in this study and their variants in ASD.

The genetics of ASD is highly complex and heterogeneous. However, Mendelian disorders such as Timothy syndrome, Joubert syndrome, Rett syndrome, and Fragile-X syndrome, and some chromosome abnormalities have ASD as a key feature [55], thereby suggesting additional candidate genes and their gene families amenable to targeted association testing in available GWAS samples. In particular, copy number variants including the genes identified in this study are associated with a number of phenotypic findings, including autism, as listed in Table 3. Complex disorders that are likely to share some risk genes, such as schizophrenia and bipolar disorder, also provide a substantial list of potential candidates for analysis [36]. Here, we sought to investigate the role of CCG selected because they are enriched in brain, associated with idiopathic ASD, and relevant to Mendelian disorders such as TS that feature ASD or complex diseases such as bipolar disorder. As both L-type and T-type calcium channel genes exhibited association with ASD, it is likely that subtype-specific abnormal activities of Ca^2+^ channels could affect distinct neuronal functions. Sequencing of the genes identified in this study and functional studies of the linked polymorphisms may further expand our understanding of the recurring association of autism and calcium channel function.

**Table 3:** Copy number variants encompassing associated SNPs

**Channel/Gene symbol**	**SNP**	**Numbers of deletions/duplications**^**a**^	**Relevant CNV phenotypes**
Ca_v_1.2	rs10848653	12/13	Autism, developmental delay, speech delay
CACNA1C			
Ca_v_3.1	rs198538	3/3	Microcephaly, mental retardation/developmental delay
CACNA1G	rs198545	3/2	Microcephaly, Mental retardation/developmental delay
Ca_v_3.3	rs5750860	0/10	Mental retardation/developmental delay
CACNA1I			

^a^Reported from two consortia: ISCA (International Standards for Cytogenomic Arrays) reports pathogenic CNV and DECI (Database of Chromosomal Imbalance and Phenotype in Humans Using Ensembl Resources) reports chromosomal imbalance in patients

(http://genome.ucsc.edu/cgibin/hgc?hgsid=290095779&c=chr12&o=565412&t=3707025&g=decipher&i=252381).

## Conclusions

Biological plausibility and genetic support for the role of CCG in ASD led us to conduct the current targeted association study with GWAS data from AGP and AGRE. Four associations in three CCGs provide evidence of a role for common alleles in CCGs as predisposing risk factors for ASD. Follow-up studies of other candidate CCGs may reveal a more complete picture of the role of the CCG in ASD. CCG sequencing studies of ASD probands can be used to assess the role of rare variants in these genes.

## Abbreviations

ASD: Autism spectrum disorders; ADI-R: Autism Diagnostic Interview; CCG: Calcium channel gene; FBAT: Family Based Association Test; GWAS: Genome-wide Association Study; MDS: Multi-dimensional scaling.

## Competing interests

The authors declare no competing interests.

## Authors’ contributions

ATL participated in the conception and design of the study, performed the statistical analysis, and wrote the first draft of the manuscript. XD contributed to statistical analysis. JAMA provided the biological, CNV, and disease interpretation of the study, and contributed to the writing of the manuscript. RMC participated in the conception, design, and coordination of the study, and wrote the final draft of the manuscript. All authors read and approved the final manuscript.

## Web resources

AGRE, http://www.agre.org/index.cfm, AGP, http://www.autismgenome.org/, CDC news, http://www.cdc.gov/media/releases/2012/p0329_autism_disorder.html, FBAT software, http://www.biostat.harvard.edu/~fbat/default.html, GENSAT, http://www.gensat.org/search.jsp, HBT, http://hbatlas.org/pages/hbtd, HUGO Gene Nomenclature Committee, http://www.genenames.org/, IMPUTE2, http://mathgen.stats.ox.ac.uk/impute/impute_v2.html, MaCH, http://www.sph.umich.edu/csg/abecasis/MACH/tour/imputation.html, PLINK software, http://pngu.mgh.harvard.edu/~purcell/plink/dataman.shtml#extract, Tfsitescan, http://www.ifti.org/cgi-bin/ifti/Tfsitescan.pl, UCSC Genome Browser, http://genome.ucsc.edu/cgi-bin/hgGateway.
